# Adsorption Kinetics: Classical, Fractal, or Fractional?

**DOI:** 10.1021/acs.langmuir.5c01726

**Published:** 2025-07-21

**Authors:** Evangelos Bakalis, Francesco Zerbetto

**Affiliations:** Dipartimento di Chimica “G. Ciamician”, Università di Bologna, V. P. Gobetti 85, 40129 Bologna, Italy

## Abstract

Adsorption-limited kinetics may be described by phenomenological pseudo-order models. Such models leverage on the general principle that the rate of change of the adsorbed material depends on some power of its concentration, and their solutions provide the quantity of adsorbed molecules per unit mass of the sorbent material as a function of time. The assumptions made about how the solute molecules (adsorbents) are distributed around the sorbent material and whether or not diffusion effects are present are crucial for defining the rate of change. In the first case, the homogeneous uniform distribution of solute molecules and the absence of diffusion effects are well-described by classical modeling (integer-order derivatives). In the second case, fractal modeling arises from a departure from homogeneous uniform distribution, time is apparently contracted, and diffusion effects are still absent. In the third case, deviation from both conditions leads to fractional modeling; unlike fractal modeling, there are memory effects that exert an action on a limited number of process steps. We present briefly solutions for various classical and fractal kinetic models that describe adsorption. For the first time, we present adsorption kinetics under the framework of fractional calculus. In particular, we provide detailed expressions for pseudo-first-order fractional kinetics, while for higher orders, recursive relations amenable to numerical treatment are given. Application of each model is discussed.

## Graphical abstract

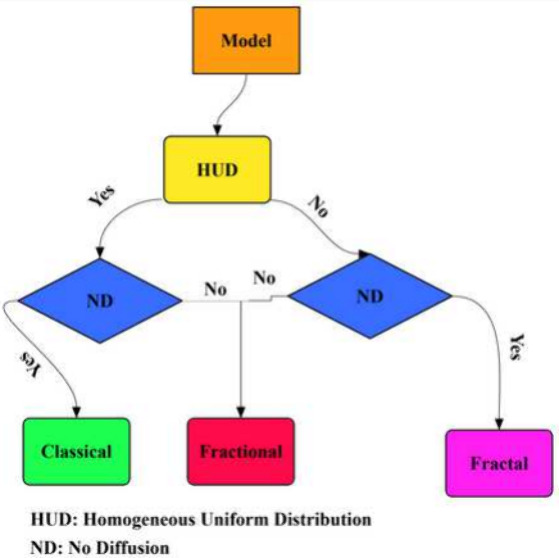

## Introduction

A rate equation is a phenomenological relationship that establishes a link between a quantity and its rate of change. It is converted into an equation by adding appropriate parameters, which are typically considered constant, ensuring conservation of units of measurement. The construction of such an equation from raw data is often a challenge.^1^ In chemistry, reaction kinetics can be modeled by rate equations and describe a variety of processes from catalysis^2^ to enzymatic reactions^3^ to sorption,^4^ just to name a few. Adsorption is a subcategory of sorption since it describes the process by which atoms, ions, or molecules adhere to a surface and form a monolayer film as a result of successive adhesion events. The adsorbates, are the substances that adhere to the surface (adsorbent or sorbent) from gases, solutions, or dissolved solids, never seep into the bulk material of the surface.^5^ There are two main categories of adsorption that can be identified. The first one, generally referred to as physisorption, is driven by van der Waals forces and has comparatively weak adsorption energy. Langmuir was the first to consider the second kind of adsorption, which is referred to as chemisorption and is sustained by covalent forces.^6^ The two types may coexist and creation of multiple adsorbate layers can also occur.^5^

In what follows, we will describe adsorption limited-kinetics although the models that will be used are not restricted to it, but can be applied in any other chemical process. Both classical and fractal calculus can be used for modeling, depending on the initial assumptions, and, in particular, the distribution of the solute molecules near the solid surface. Diffusion is not taken into account in any form. Furthermore, modeling can be done within the framework of fractional kinetics if we assume that the rates of adsorption and desorption carry memory. The reason for the choice of adsorption is 2-fold; on the one hand, the constructed equations are simpler, and a direct comparison between them for classical, fractal, and fractional kinetics can be made. On the other hand, adsorption is linked to a wide range of physical, chemical, biological, and environmental phenomena and/or technological applications. Adsorption-based systems are widely used in applied technology, which is not surprising given that adsorption-based techniques are simple to use, economical, and energy-efficient. A tangible example of great importance nowadays is water remediation where removal of pollutants from wastewater is accomplished with the use of adsorbents.^7^ To maximize their effectiveness in eliminating contaminants and other impurities from a system, sorbents and adsorbates must be designed with an understanding of their kinetics and thermodynamic characteristics. The Langmuir isotherm for monolayer formation is the most widely used model for studying the thermodynamics of surface adsorption.^6^

Adsorption kinetics is frequently studied within the framework of the so-called pseudo-*n*th-order equations, where the pseudo-first-order was proposed more than a century ago by Lagergren.^8^ In addition, pseudo-second-order equations have been proposed.^9–13^ When the distribution of solute molecules around the sorbent material departs from homogeneously uniform, the classical pseudo-first-order kinetics is forfeited and fractal pseudo-first-order kinetics emerge.^14^ Fractal kinetics modeling has been extended to higher orders.^15^

In the present work, we present the solutions of various pseudo-*n*th-order models that describe adsorption-limited kinetics either using classical or fractal kinetics. For the very first time, to the best of our knowledge, we present adsorption kinetics in the framework of fractional kinetics, and we provide analytical solutions for pseudo-first-order fractional kinetics as well as recursive relations amenable to numerical treatment for higher orders. Fractional kinetics allows adsorption and desorption rates to include memory effects. Such effects arise either because diffusive processes evolve at short time scales^16^ or because processes become irreversible as the system passes various metastable states and dissipates energy. In this case, memory is the convolution of the rate with the instantaneous concentration.

## Adsorption Kinetics and Pseudo-*n*th-Order Equations

An adsorption kinetics-limited process in a solid–liquid interface is one in which no diffusion effects are taken into account, and therefore the process can be represented by two states; in the first one, a finite number of adsorbates (molecules from the liquid phase) and adsorbents (available sites on the solid) coexist, and the second one contains only the complex conjugates. In addition, continuous stirring, for instance, can ensure a uniform distribution of solute molecules in front of the sorbent. The kinetics are represented by a reversible reaction given by eq 1

1 $$A+{\;}^{{}^{\circ}}\overset{k_{a}}{\underset{k_{d}}{⇄}}A\left({\;}^{{}^{\circ}}\right)$$

where *A* stands for the solute molecules contained in volume *V* in contact with the sorbent of mass *m*. The symbol ° stands for the empty sites on the surface of the sorbent material whose number is limited, and *A*(°) the complex conjugate or adsorbed molecules. *k*_a_ and *k*_d_ are the adsorption and desorption rates, respectively, in units of *T* ^–1^. It is possible to formulate a reversible first-order rate expression for the reaction described by eq 1 using mass-balanced equations for solution concentration.^17^ Though, it is more convenient the use a phenomenological model,^8,9,11,17^ whose general form implies that the concentration of the solute molecules at time *t* is proportional to a power *n* of the concentration of solute molecules being adsorbed at time *t* ^5^

2 $$\frac{dC\left(t\right)}{dt}=-k_{n,c}{\left(C_{0}-C\left(t\right)\right)}^{n}$$

where *C*(*t*) and *C*_0_ = *C*(*t* = 0) are the concentrations of the solute molecules at time *t* and time *t* = 0, respectively, and *k*_*n*,c_ is the rate constant expressed in proper units. An equation that gives the number of adsorbed molecules for a given solution of volume (*V*) in contact with sorbent of mass (*m*) at time *t* would be preferable than studying eq 2. Such an equation will also describe a specific sorbent material capacity to adsorb solute molecules. We define the function $g\left(t\right)=\frac{V}{m}\left(C_{0}-C\left(t\right)\right)$ in units of $\frac{\mathrm{mg}}{g}$ (mg of adsorbed material per g of sorbent) that takes the extreme values *g*(*t* = 0) = 0, and $\underset{t\to \infty }{\lim}g\left(t\right)=g_{e}$ (value at equilibrium). In addition, through the function *g*(*t*) the amount of adsorbate per unit mass of adsorbent is defined as $F=\frac{g\left(t\right)}{g_{e}}$ . Moreover, the surface coverage, $\theta \left(t\right)=\frac{M_{w}}{g_{m}}g\left(t\right)$ , is expressed as a function of *g*(*t*), where *g*_m_ is the maximum capacity of the sorbent, which indicates the amount (*g*) of adsorbed per gram of sorbent, and *M*_w_ is the solute’s molar weight (g/mol). We can therefore determine the amount of adsorbate per unit mass of adsorbent and the surface coverage by knowing the explicit form of *g*(*t*) over time. Equation 2 in terms of the function *g*(*t*) reads

3 $$\frac{dg\left(t\right)}{dt}=k_{n}{\left(g_{e}-g\left(t\right)\right)}^{n}$$

with *k*_*n*_ is the overall rate constant in units of $\frac{1}{s}{\left(\frac{g}{\mathrm{mg}}\right)}^{n-1}$ and *n* is the order of the model. Equation 3 is a Riccati-type differential equation and can be easily solved. By setting *y*(*t*) = *g*_e_ – *g*(*t*) and *x*(*t*) = *y*(*t*)^1–*n*^ we find

4 $$g\left(t\right)=g_{e}-g_{e}{\left(1-g_{e}^{n-1}k_{n}\left(1-n\right)t\right)}^{1/1-n}$$

Equation 4 is the general solution for any pseudo-*n*th-order classical model that describes sorption processes with *n* ≥ 0. Its form is similar to *q*-exponential distribution, $P_{q}\left(x\right)∼{\left(1-\left(1-q\right)\frac{x^{2}}{k_{B}T}\right)}^{1/1-q}$ , which finds use in Tsallis’ statistics,^18^ and for *q* = 1 returns the classical exponential function. For *n* = 1, eq 3 becomes $\frac{dg\left(t\right)}{dt}=k_{1}\left(g_{e}-g\left(t\right)\right)$ . It was first introduced by Lagergren^8^ and its solution is *g*(*t*) = *g*_e_(1 – e^–*k_1_t*^), see also Table 1, where detailed solutions for each model mentioned in this work are listed. Equation 3 for *n* = 2 was discussed in detail in^9,19–21^ and describes a pseudo-second-order model whose linearized solution is given by eq 5.

5 $$\frac{1}{g\left(t\right)}=\frac{1}{g_{e}}+\frac{1}{g_{e}^{2}k_{2}t}$$

In the literature, pseudo-first- and -second-order kinetics are frequently employed to analyze experimental data. This type of kinetics became very popular, especially after the work of Ho and McKey,^9^ who analyzed several data sets from literature and concluded that the pseudo-second-order kinetics always provides the best correlation of the experimental data. Recent research has challenged this widely held notion,^22^ emphasizing the necessity of cautious statistical analysis because *R* ^2^ is not a model’s sole trustworthy quality index.

**Table 1:** Overview of a Number of Pseudo-Order Equations That Describe Adsorption-Limited Kinetics Using Fractional, Fractal, and Classical (Integer Order Derivatives) Kinetics, Together with Their Solutions and Linearised Versions When Available^a^

order	equation	solution	linearized form
Classical Kinetics			
zeroth	$\frac{dg\left(t\right)}{dt}=k_{0}$	*g*(*t*) = *k*_0_ *t*	*g*(*t*) = *k*_0_ *t*
first	$\frac{dg\left(t\right)}{dt}=k_{1}\left(g_{e}-g\left(t\right)\right)$	*g*(*t*) = *g*_e_(1 – e^–*k*_1_ *t*^)	ln(*g*_e_ – *g*(*t*)) = ln(*g*_e_) – *k*_1_ *t*
second	$\frac{dg\left(t\right)}{dt}=k_{2}{\left(g_{e}-g\left(t\right)\right)}^{2}$	$g\left(t\right)=\frac{g_{e}^{2}k_{2}t}{1+g_{e}k_{2}t}$	$\frac{1}{g\left(t\right)}=\frac{1}{g_{e}}+\frac{1}{g_{e}^{2}k_{2}t}$
*n*th	$\frac{dg\left(t\right)}{dt}=k_{n}{\left(g_{e}-g\left(t\right)\right)}^{n}$	$g\left(t\right)=g_{e}-g_{e}{\left(1-g_{e}^{n-1}k_{n}\left(1-n\right)t\right)}^{1/1-n}$	${\left(1-\frac{g\left(t\right)}{g_{e}}\right)}^{1-n}=1-\frac{k_{n}\left(1-n\right)}{g_{e}^{1-n}}t$
mixed	$\frac{dg\left(t\right)}{dt}=\overset{2}{\underset{n=1}{\sum }}k_{n}{\left(g_{e}-g\left(t\right)\right)}^{n}$	$g\left(t\right)=g_{e}\frac{\left(1-e^{-k_{1}t}\right)}{1-\frac{k_{2}g_{e}}{k_{1}+k_{2}g_{e}}e^{-k_{1}t}}$	
Fractal Kinetics			
zeroth	$\frac{dg\left(t\right)}{dt}=ak_{0}t^{a-1}$	*g*(*t*) = *k*_0_ *t* ^*a*^	ln(*g*(*t*)) = ln(*k*_0_) + *a* ln(*t*)
first	$\frac{dg\left(t\right)}{dt}=ak_{1}t^{a-1}\left(g_{e}-g\left(t\right)\right)$	*g*(*t*) = *g*_e_(1 – e^–*k*_1_ *t* ^*a*^^)	ln(*g*_e_ – *g*(*t*)) = ln(*g*_e_)– *k*_1_ *t* ^*a*^
second	$\frac{dg\left(t\right)}{dt}=ak_{2}t^{a-1}{\left(g_{e}-g\left(t\right)\right)}^{2}$	$g\left(t\right)=\frac{g_{e}^{2}k_{2}t^{a}}{1+g_{e}k_{2}t^{a}}$	$\frac{1}{g\left(t\right)}=\frac{1}{g_{e}}+\frac{1}{g_{e}^{2}k_{2}t^{a}}$
*n*th	$\frac{dg\left(t\right)}{dt}=ak_{n}t^{a-1}{\left(g_{e}-g\left(t\right)\right)}^{n}$	$g\left(t\right)=g_{e}-g_{e}{\left(1-g_{e}^{n-1}k_{n}\left(1-n\right)t^{a}\right)}^{1/1-n}$	${\left(1-\frac{g\left(t\right)}{g_{e}}\right)}^{1-n}=1-\frac{k_{n}\left(1-n\right)}{g_{e}^{1-n}}t^{a}$
mixed	$\frac{dg\left(t\right)}{dt}=at^{a-1}\overset{2}{\underset{n=1}{\sum }}k_{n}{\left(g_{e}-g\left(t\right)\right)}^{n}$	$g\left(t\right)=g_{e}\frac{\left(1-e^{-k_{1}t^{a}}\right)}{1-\frac{k_{2}g_{e}}{k_{1}+k_{2}g_{e}}e^{-k_{1}t^{a}}}$	
Fractional Kinetics			
zeroth	Dta0Cg(t)=k0	$g\left(t\right)=\frac{k_{0}}{\Gamma \left(1+a\right)}t^{a}$	$\ln\left(g\left(t\right)\right)=\ln\left(\frac{k_{0}}{\Gamma \left(1+a\right)}\right)+a\ln\left(t\right)$
first	Dta0Cg(t)=k1(ge−g(t))	$g\left(t\right)=g_{e}\left(1-E_{a}\left(-k_{1}t^{a}\right)\right)$	$\frac{g_{e}-g\left(t\right)}{g_{e}}=E_{a}\left(-k_{1}t^{a}\right)$
second	Dta0Cg(t)=k2(ge−g(t))2	$g\left(t\right)=\frac{k_{2}g_{e}^{2}}{\Gamma \left(1+a\right)}t^{a}-2\frac{k_{2}^{2}g_{e}^{3}}{\Gamma \left(1+2a\right)}t^{2a}$	
mixed	Dta0Cg(t)=∑n=12kn(ge−g(t))n	$g\left(t\right)=\frac{g_{e}\left(k_{1}+k_{2}g_{e}\right)}{\Gamma \left(1+a\right)}t^{a}-\frac{g_{e}\left(k_{1}+k_{2}g_{e}\right)\left(k_{1}+2k_{2}g_{e}\right)}{\Gamma \left(1+2a\right)}t^{2a}$	

a For classical modeling, the rates *k*_*n*_ are considered constant. They are transformed to *k̅*_*n*_ = *at* ^*a*–1^ *k*_*n*_ exhibiting thus time dependence for fractal kinetics. For fractional kinetics, there is also time dependence whose effect at the time *t* is expressed via the convolution of the rate with *g*(*t*). For fractional kinetics and for *n* > 1, analytical solutions do not exist and consequently are not displayed in Figures 3 and 4.

A direct extension of the pseudo-first- and -second-order kinetics is a mixed pseudo-first- and -second-order model that is described by

6 $$\frac{dg\left(t\right)}{dt}=\overset{2}{\underset{n=1}{\sum }}w_{n}k_{n}{\left(g_{e}-g\left(t\right)\right)}^{n}$$

where *w*_*n*_ is the weight coefficient. For *w*_1_ = *w*_2_ = 1, eq 6 has been derived either by first-principles,^11^ or by Taylor expansion around the equilibrium point.^23^ In order to solve eq 6, we define the function $y\left(t\right)=\ln\left(\frac{g_{e}-g\left(t\right)}{g_{e}-g\left(t\right)+\lambda }\right)$ , where $\lambda =\frac{w_{1}k_{1}}{w_{2}k_{2}}$ , and taking the derivative, we arrive at $d\ln\left(\frac{g_{e}-g\left(t\right)}{g_{e}-g\left(t\right)+\lambda }\right)=-w_{1}k_{1}t$ , whose solution is known. Upon rearranging the terms, we find

7 $$g\left(t\right)=g_{e}\frac{1-e^{-w_{1}k_{1}t}}{1-\frac{g_{e}}{g_{e}+\frac{w_{1}k_{1}}{w_{2}k_{2}}}e^{-w_{1}k_{1}t}}$$

see also Table 1, where eq 7 for *w*_1_ = *w*_2_ = 1 is listed. In contrast to low concentrations, where the second order plays a significant role, the pseudo-first-order equation dominates for high initial concentrations of solute molecules.^11^

## Fractal Kinetics and Adsorption

The fractal approach of the adsorption process constitutes a direct generalization of the classical pseudo-*n*-order equations. Kopelman, in his seminal work about fractal reaction kinetics, assumed that the reaction rate, which was once thought to be constant, has a time dependence of the form *k* = *k*_1_ *t* ^–*h*^, with 0 ≤ *h* ≤ 1 for (*t* > 1).^14^ This time variation is equivalent to a shrinking of time.

### Shrinking of Time

The issue is what can cause the “shrinking” of time, or, in other words, what kind of variations in the distribution of solute molecules can give rise to the presence of *k*(*t*). We start by assuming two identical containers where exactly the same experiment is carried out and the only difference is the preparation time. There is a good chance that identical values for a measurement in the two containers will occur. Nevertheless, the time this measurement is taken in relation to the preparation time differs for the two containers: *t*_1_ for the first and *t*_2_ for the second, with *t*_1_ > *t*_2_. We determine that *k*_1_ < *k*_2_ from the definition of *k*_*n*_ = *kt*_*n*_^–*h*^ for *n* = 1, 2. This relation reflects changes in the distribution of the solute molecules around the sorbent as the time passes and does not imply any memory effects related to the concentrations.

A classical pseudo-order equation is formed under the implicit assumption that the concentration of the solute molecules is uniformly random and remains random, for instance under stirring. A fractal-like adsorption indicates that an initially uniformly random distribution tends to become more ordered over time, even in the presence of stirring. So, a kind of self-organization is imposed on the solute molecules, whose origin must be sought in the structure of the sorbent material. Assuming the system is at equilibrium, thermal noise causes solute molecules to move randomly prior to being adsorbed. It is known that for a 1-dimensional (1D) random walk, the probability of returning to its origin is 1, goes down to 0.34 for a 2-dimensional (2D) random walk, and 0 (it never returns to the origin) for a 3-dimensional (3D) random walk.^24^

In solution, solute molecules perform a 3D walk. This is true also around the sorbent material and classical pseudo-*n*-order equations can be applied for extracting insights. As time progresses, the molecules organize around the surface and the walk can reduce to 2D and even 1D. Return to the initial position makes the motion of the molecules akin to a vibration, rather than pure diffusion. This is the case where pseudofractal-like kinetics is in operation. A number of models have been put forth to explain fractal-like adsorption kinetics. Haerifar and Azizian described a mechanism that might result in time-dependent rate coefficients and fractal-like kinetics for both homogeneous^25^ and nonhomogeneous surfaces.^26^ The different paths a solute molecule must take to be adsorbed by a host site at a homogeneous or heterogeneous surface are taken into consideration by the model. Furthermore, when a solute molecule is close to a host site, the site may already be occupied. This results in additional wandering, which adds to the rate constant final time dependence when viewed from the standpoint of an ensemble average. Hu et al.^27^ presented some well-established models^28–30^ by changing the rate constant to a time-dependent one according to Kopelman’s suggestion, and the findings adapt well data originating from adsorption in bed-column.

These models lack a mathematical explanation that results in a rate coefficient of the power-law type. The same result discussed above can be reached starting from the classical pseudo-*n*-order model described by eq 3. Considering that time has been subject to scaling, i.e. fractal time, so from *t* we go to *t* ^*a*^ with *a* being the fractal exponent. The l.h.s of eq 3 is written as $\frac{dg\left(t\right)}{{dt}^{a}}$ , which is also written as $\frac{dg\left(t\right)}{dt}\frac{dt}{{dt}^{a}}$ and by substituting the derivative $\frac{dt}{{dt}^{a}}=\frac{t^{1-a}}{a}$ , we end up with eq 8

8 $$\frac{dg\left(t\right)}{dt}=ak_{n}t^{a-1}{\left(g_{e}-g\left(t\right)\right)}^{n}$$

The rate coefficient *k*_*n*_ of eq 3 changes to an effective one *at* ^*a*–1^ *k*_*n*_ in eq 8, where the exponent *h* of Kopelman’s definition is equal to *h* = 1 – *a*, as can be seen by comparing the terms *t* ^–*h*^ and *t* ^*a*–1^. Equation 8 is again a Riccati-type of differential equation and its solution reads

9 $$g\left(t\right)=g_{e}\left\{1-{\left(1-\left(1-n\right)g_{e}^{n-1}k_{n}t^{a}\right)}^{1/1-n}\right\}$$

Brouers and Sotolongo-Costa derived eq 9 and presented it as a universal solution for the kinetics of complex systems with power-law and/or stretched exponential behaviors.^15^ The dynamics for pseudo-first-order, -second-order, and higher order fractal-like equations can be easily extracted by eq 9, see the corresponding solutions in Table 1. A mixed pseudo-first- and -second-order fractal equation can be written, similar to eq 6, as

10 $$\frac{dg\left(t\right)}{dt^{a}}=\overset{2}{\underset{n=1}{\sum }}w_{n}k_{n}{\left(g_{e}-g\left(t\right)\right)}^{n}$$

which is solved in a similar manner to eq 6, and its solution reads

11 $$g\left(t\right)=g_{e}\frac{1-e^{-w_{1}k_{1}t^{a}}}{1-\frac{g_{e}}{g_{e}+\frac{w_{1}k_{1}}{w_{2}k_{2}}}e^{-w_{1}k_{1}t^{a}}}$$

see also Table 1, where the solution of eq 11 for *w*_1_ = *w*_2_ = 1 is listed.

Experimental data have been fitted using pseudo-first-order fractal kinetics. It is worth mentioning that eq 9 for *n* = 1, pseudofractal first order, contains the Avrami function,^31^ which was utilized to demonstrate how chitosan membranes work as an adsorbent to remove Hg­(II) from aqueous solutions.^32^ Moreover, the adsorption of methylene blue dye from amphoteric and amphiphilic PCL-*co*-PHEMA hydrogel is accurately predicted by pseudo-first-order fractal kinetics.^33^

## Adsorption and Fractional Kinetics

Fractional calculus is the extension of the standard calculus theory to non-integer order derivatives and integrals. This field of mathematics started in 1695 when de L’Hospital asked Leibniz what d^1/2^/d*x* ^1/2^ might mean. Fractional calculus was the subject of study in pure mathematics for the next two centuries. By defining fractional operators and researching their key characteristics, Euler, Fourier, Abel, Liouville, Riemann, and Hadamard, among others, laid the groundwork for this field. In the last few decades, fractional calculus has gained popularity due to its versatility for describing a variety of natural phenomena, offering valuable insights into intricate systems.^34,35^ Essential components of fractional calculus are its operators (derivative and integrals), which are nonlocal operators allowing thus incorporation of memory effects, or, in other words, describing how the present is affected to a certain extent by the past.

Fractional kinetics describes nonlocal variations. The nonlocality occurs because there are no dynamic processes operating at infinite speeds. There exists a temporal delay between the causes and the outcomes. For instance, in modeling chemical reactions, one considers, to a first approximation, that the reaction rates involved at the various stages of the reaction are constant. Nevertheless, in many cases, the measurements do not match the modeling outputs, and the system’s non-Markovian behavior is likely caused by reaction rates that carry some memory of the previous events.^36,37^ Moreover, the reaction rates may have some memory due to diffusive dynamics, which are not considered in adsorption limited-kinetics. While the solute molecule will move randomly until it is adsorbed, a successful event (adsorption) requires time, which is known in the literature as the mean first time passage.^38^

Fractional derivatives are not defined in a unique way. The Caputo fractional derivative is defined as

12 Dta0Cf(t)=1Γ(n−a)∫0tf(n)(τ)(t−τ)n−1−a⁡dt,⁣n−1<a<n

with *n* = 1 for 0 < *a* < 1, while eq 12 reduces to classical derivative for *a* = 1. In addition, the Riemann–Liouville operator is a well-established fractional derivative that reads

13 Dta0RLf(t)=1Γ(n−a)dndtn∫0tf(τ)(t−τ)n−1−a⁡dt,⁣n−1<a<n

Equations 12 and 13 are interrelated as

14 Dta0Cf(t)=Dta0RLf(t)−f(0)Γ(1−a)t−a

The application of the Caputo fractional derivative on simple functions, let say of the form *f*(*t*) = *t* ^*b*^, gives

15 Dta0Ctb=Γ(1+b)Γ(1+b−a)tb−a

Every single kinetic model can be generalized and expressed by fractional derivatives and/or integrals, a unified fractional kinetic description.^39^ The fractional version of eq 3 reads

16 Dta0Cg(t)=kn(ge−g(t))n

Equation 16 for *n* = 0 has the form $\frac{1}{\Gamma \left(1-a\right)}\int_{0}^{t}{\left(t-\tau \right)}^{-a}ġ\left(\tau \right)dt=k_{0}$ and is called zeroth-order fractional kinetics. To solve it, we use the Laplace pair, *L*{*f*(*t*)}­(*s*) = ∫_0_^∞^e^–*st*^ *f*(*t*)­d*t*, which converts it to $s^{-1+a}\left(sg\left(s\right)-g\left(0\right)\right)=\frac{k_{0}}{s}$ , with *g*(0) = 0. When the latter is inverted into the time domain, the pseudo fractional zeroth-order equation’s solution reads.

17 $$g\left(t\right)=\frac{k_{0}}{\Gamma \left(1+a\right)}t^{a}$$

It is important to note that the solution for fractal kinetics of the pseudo-zeroth order is similar to eq 17. The factor Γ­(1 + *a*) that is present in the case of fractional kinetics modeling is the only difference.

Equation 16 for *n* = 1 has the form $\frac{1}{\Gamma \left(1-a\right)}\int_{0}^{t}{\left(t-\tau \right)}^{-a}ġ\left(\tau \right)dt=k_{1}\left(g_{e}-g\left(t\right)\right)$ and is called first-order fractional kinetics. Once more using the Laplace pair, we write $g\left(s\right)=\frac{k_{1}g_{e}}{s\left(s^{a}+k_{1}\right)}$ . Simplifying the denominator $\frac{1}{s\left(s^{a}+k_{1}\right)}=\frac{1}{k_{1}}\left(\frac{1}{s}-\frac{1}{s^{a}+k_{1})}\right)$ and making use of $L\left\{t^{b-1}E_{a,b}^{n}\left(\pm \lambda t^{a}\right)\right\}\left(s\right)=\frac{s^{an-b}}{{\left(s^{a}\mp \lambda \right)}^{n}}$ ,^40^ we arrive at

18 $$g\left(t\right)=g_{e}-g_{e}E_{a}\left(-k_{1}t^{a}\right)$$

where *E*_a_(−*k*_1_ *t* ^*a*^) is the one parameter Mittag-Leffler function, which returns the exponential function for *a* = 1 and has the form $E_{a}\left(x\right)=\overset{\infty }{\underset{m=0}{\sum }}\frac{x^{m}}{\Gamma \left(1+am\right)}$ .^41,42^ In addition, the function $E_{a,b}^{n}\left(\pm \lambda t^{a}\right)=\frac{1}{\Gamma \left(m\right)}\overset{\infty }{\underset{m=1}{\sum }}\frac{\Gamma \left(n+m\right)}{m!\Gamma \left(am+b\right)}z^{m}$ is the three parameter Mittag-Leffler.^40,43^

Equation 16 for *n* = 2 is a Riccati type fractional differential equation with the form $\frac{1}{\Gamma \left(1-a\right)}\int_{0}^{\infty }{\left(t-\tau \right)}^{-a}ġ\left(\tau \right)d\tau =k_{2}{\left(g_{e}-g\left(t\right)\right)}^{2}$ and is called second-order fractional kinetics. A power series can be used to express the latter’s solution. First, we define the function ϕ­(*t*) = *g*_e_ – *g*(*t*) with initial condition ϕ(0) = *g*_e_. Taking into account that the Caputo fractional derivative of a constant is zero, a pseudo-second-order fractional equation of describing adsorption takes the form _0_^C^ *D*_*t*_^*a*^ϕ­(*t*) = – *k*_2_ϕ­(*t*)^2^. Moreover, we consider that the solution of the latter has the form ϕ­(*t*) = ∑_*n* = 0_^∞^ *b*_*n*_ *t* ^*na*^, with *b*_0_ = *g*_e_ due to the initial condition ϕ(0) = *g*_e_. By combining eqs 14 and 15, we write

19 $$\overset{\infty }{\underset{n=1}{\sum }}b_{n}\frac{\Gamma \left(1+na\right)}{\Gamma \left(1+\left(n-1\right)a\right)}t^{a\left(n-1\right)}=-k_{2}\overset{\infty }{\underset{n=0}{\sum }}\overset{n}{\underset{m=0}{\sum }}b_{m}b_{n-m}t^{an}$$

where the double summation at the r.h.s of eq 19 is the Cauchy product of two time series. Rearranging the terms in eq 19 we end up with

20 $$\overset{\infty }{\underset{n=0}{\sum }}\left\{b_{n+1}\frac{\Gamma \left(1+\left(n+1\right)a\right)}{\Gamma \left(1+na\right)}+k_{2}\overset{n}{\underset{m=0}{\sum }}b_{m}b_{n-m}\right\}t^{an}=0$$

We compute the different terms *b*_*n*_ by equating terms of the same order, which up to third order have the form: $b_{1}=-\frac{k_{2}g_{e}^{2}}{\Gamma \left(1+a\right)}$ , $b_{2}=2\frac{k_{2}^{2}g_{e}^{3}}{\Gamma \left(1+2a\right)}$ , and $b_{3}=-4\frac{k_{2}^{3}g_{e}^{4}}{\Gamma \left(1+3a\right)}\left(1+\frac{1}{4}\frac{\Gamma \left(1+2a\right)}{\Gamma {\left(1+a\right)}^{2}}\right)$ . For *n* = 2, the solution of eq 16 up to third order reads

21 $$g\left(t\right)=\frac{k_{2}g_{e}^{2}}{\Gamma \left(1+a\right)}t^{a}-2\frac{k_{2}^{2}g_{e}^{3}}{\Gamma \left(1+2a\right)}t^{2a}+4\frac{k_{2}^{3}g_{e}^{4}}{\Gamma \left(1+3a\right)}\left(1+\frac{1}{4}\frac{\Gamma \left(1+2a\right)}{\Gamma {\left(1+a\right)}^{2}}\right)t^{3a}$$

Equation 6 for *w*_1_ = *w*_2_ = 1 under the action of a Caputo fractional derivative describes a mixed first–second-order fractional kinetics, which takes the form _0_^C^ *D*_*t*_^*a*^ *g*(*t*) = ∑_*n* = 1_^2^ *k*_*n*_(*g*_e_ – *g*(*t*))^*n*^. Following similar steps as we did for the second-order fractional kinetics equation we find

22 $$g\left(t\right)=\frac{g_{e}\left(k_{1}+k_{2}g_{e}\right)}{\Gamma \left(1+a\right)}t^{a}-\frac{g_{e}\left(k_{1}+k_{2}g_{e}\right)\left(k_{1}+2k_{2}g_{e}\right)}{\Gamma \left(1+2a\right)}t^{2a}$$

Equation 22 returns eq 21 for *k*_1_ = 0. It should be noted that the pseudo-second-order or mixed-order fractional equations can be solved using nonsingular fractional operators, such as the Caputo–Fabrizio fractional operator^44^ or the Atangana–Baleanu fractional operator.^45^ However, solutions based on these operators are questionable because they do not admit the existence of a corresponding convolution integral, of which the derivative is the left-inverse, and they also fail to satisfy the fundamental theorem of fractional calculus.^46^

## Results and Discussion

### Pseudo-Zeroth-Order Kinetic Models

Pseudo-zeroth-order kinetic models consider that the rate does not depend on the concentration of the solute molecules. Such an assumption might be valid for the early stage of adsorption if we also impose high concentrations of solute molecules and vacant sites on the sorbent material.

The amount of adsorbed material up to time *t* divided by the total amount of adsorbed material (infinite time) is shown in Figure 1. By definition this quantity is between 0 and 1; however, this is not true for a pseudo-zeroth-order model because any restriction of the concentration of solute molecules is absent. Even in these circumstances, fractal and fractional modeling changes substantially the amount of the adsorbed material with respect to what the linear dependency predicts. The departure from linearity becomes stronger the lower the exponent *a*.

**Figure 1:**
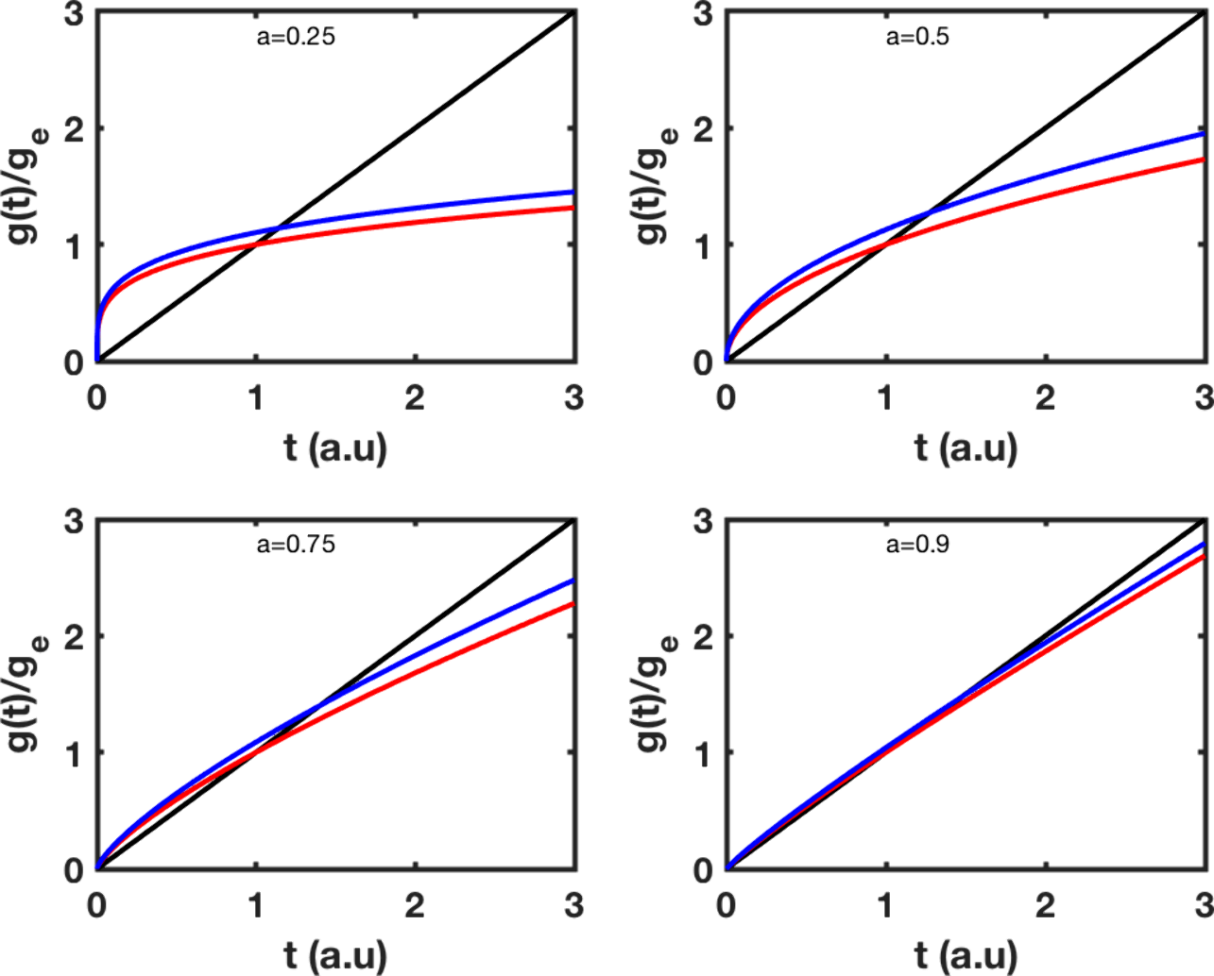
Adsorption kinetics of pseudo-zeroth-order based on fractional, fractal, and classical modeling. The rate *k*_0_ has been set to 1, and the exponent *a* takes values of 0.25, 0.50, 0.75, and 0.95. Classical kinetics, black; fractal kinetics, red; and fractional kinetics, blue.

### Pseudo-First-Order Models

Pseudo-first-order models provide a good description of three scenarios: (a) the sorbent material has few active sites relative to the available solute molecules to be adsorbed, (b) the experimental data relate to the early stage of adsorption, or (c) the initial concentration of the solute molecules is extremely high. In addition, the pseudo-first-order kinetic model is a popular one in studying the gas–solid adsorption process regulated by surface diffusion. For such a system, and based on the Langmuir isotherm, the chemical reaction is assumed to be the rate-determining step of the adsorption process on the gas–solid interface.

Figure 2 shows the ratio *g*(*t*)/*g*_e_ as a function of time, which is a solution of a classical, fractal, or fractional, pseudo-first-order equation. For early times, solutions based on fractal or fractional kinetics predict a faster adsorption rate than classical kinetics, which becomes faster as the exponent *a* becomes lower at the early stage of adsorption. Moreover, fractional kinetics with respect to the fractal and to the classical kinetics transit more rapidly to a slowly varying increase in adsorbed material. The preceding is also true for fractal dynamics, in which the transition occurs at a later time window than in the previous case. For both fractal and fractional kinetics, the adsorbed material, however, increases very slowly and tends asymptotically to the value of 1 after a crossover point, different for the two models, and it is in contrast to classical kinetics, which provides solutions that approach equilibrium the quickest. The latter clarifies how fractional and fractal kinetics differ from one another. Fractional means that a memory is formed that affects some steps of the process, opposed to fractal, where the contraction of time is a result of some constraints.

**Figure 2:**
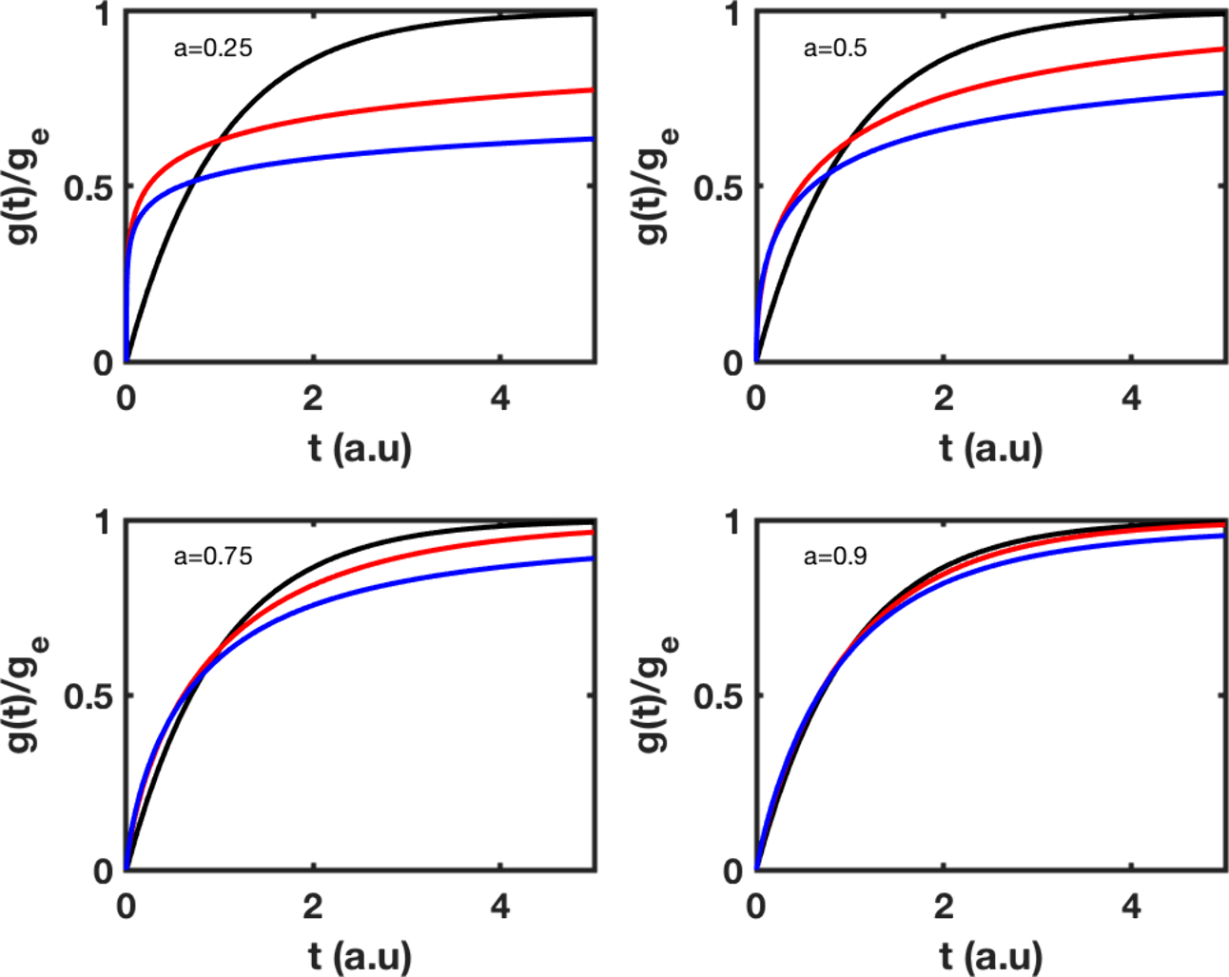
Adsorption kinetics of pseudo-first-order based on fractional, fractal, and classical modeling. The rate *k*_0_ has been set to 1, and the exponent *a* takes values of 0.25, 0.50, 0.75, and 0.95. Classical kinetics, black; fractal kinetics, red; and fractional kinetics, blue.

Either situations where the concentration of solute molecules is low, or the experiment depicts the last stages of adsorption, or the adsorbent is rich in active sites could be adequately described by a pseudo-second-order model. The pseudo-second-order kinetic model primarily depicts the gas–solid adsorption process regulated by chemical adsorption. It is well-accepted that this kind of modeling describes well chemisorption and thus a second layer of adsorption.^5^

### Pseudo-Second-Order Results

Pseudo-second-order results are displayed as a function of time in Figure 3. Solutions are offered only for fractal and classical models. The solution for fractional modeling is not presented because it is a second-order approximation (Table 1) or third-order term described by eq 21. In order to properly fit data using a solution expressed as a power series, numerical preprocessing is necessary. Beginning with eq 20, we identify the factors *b*_*n*_, and then we find the minimum number of terms that ensure solution convergence. This issue will be treated in the future. At the beginning of the adsorption process, the rate is significantly faster for fractal kinetics than for classical kinetics, the trend eventually reverses.

**Figure 3:**
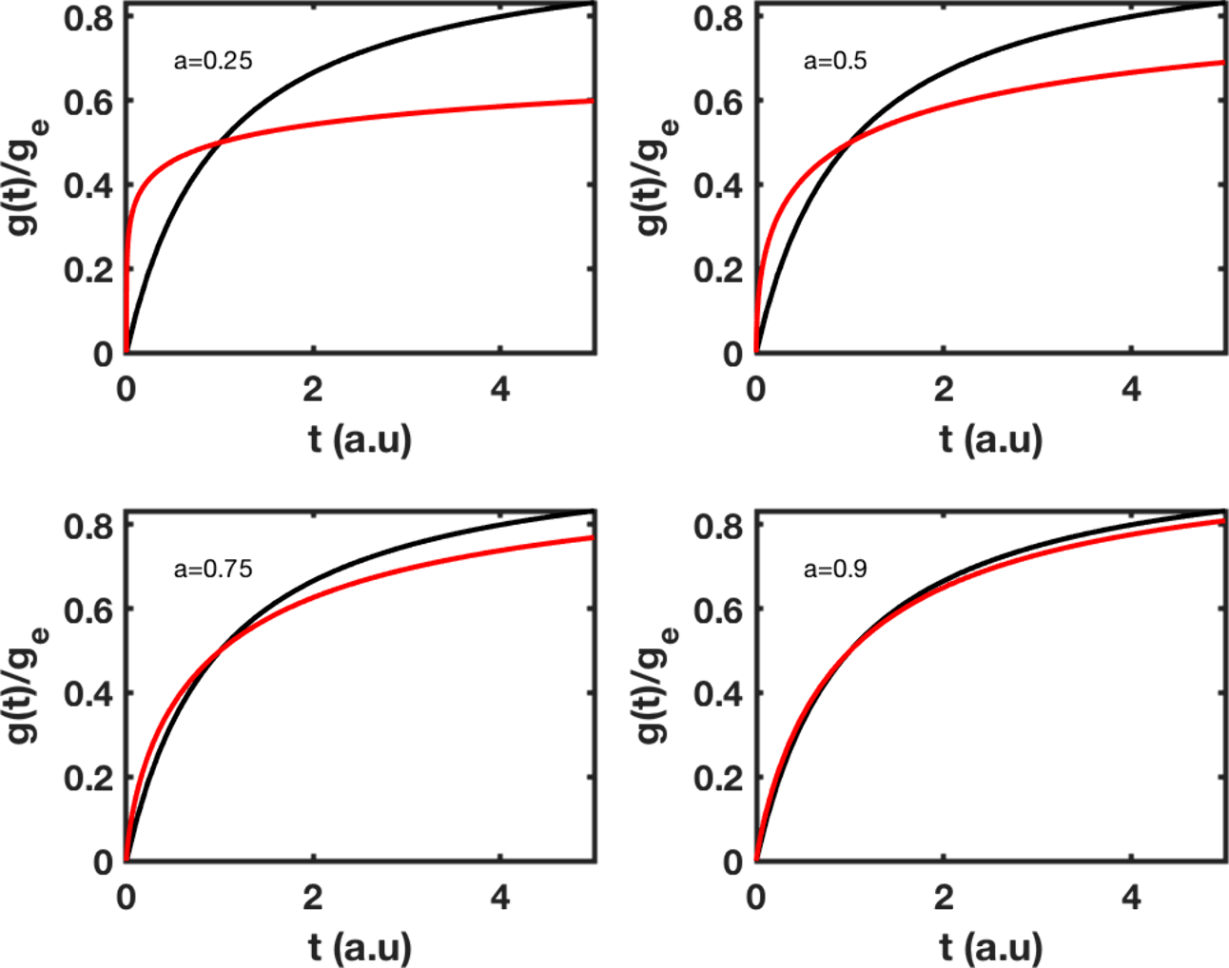
Adsorption kinetics of pseudo-second-order based on fractal, and classical modeling. The rate *k*_2_ has been set to 1, and the exponent *a* takes values of 0.25, 0.50, 0.75, and 0.95. Classical kinetics, black; for fractal kinetics, red.

### Mixed Pseudo-First–Second-Order Modeling

Mixed pseudo-first–second-order modeling is of interest because there is no assurance that a single type of adsorption adequately describes the entire process (Figure 4). It is quite possible that in different time windows, or even in the entire the adsorption process, the conditions required for the validity of the first- and second-order models coexist.

**Figure 4:**
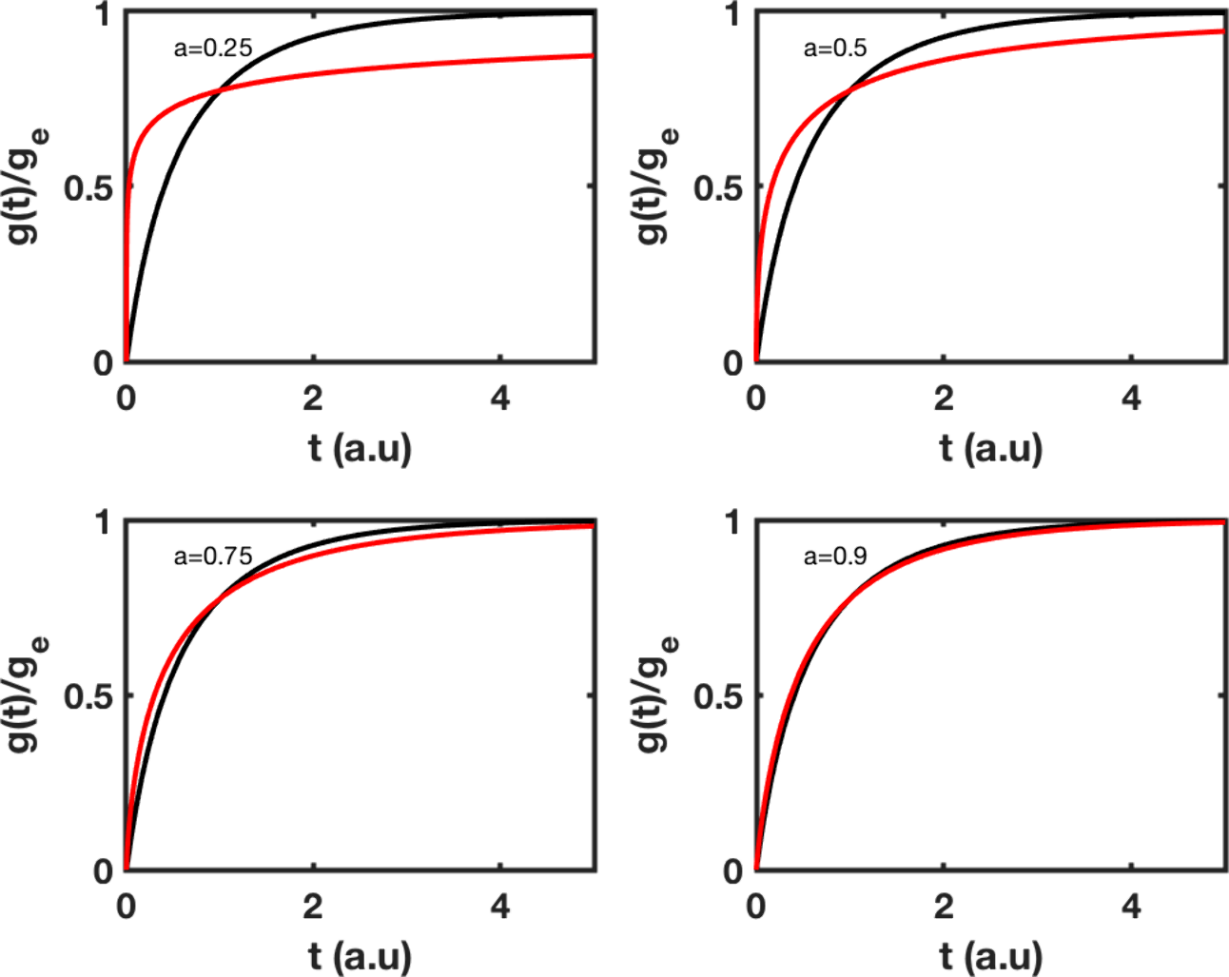
Adsorption kinetics of pseudo mixed first and second order based on fractal, and classical modeling. The rates *k*_1_ and *k*_2_ have been set to 1, and the exponent *a* accepts values of 0.25, 0.50, 0.75, and 0.95. Classical kinetics, black; fractal kinetics, red.

The rate of adsorption at the early stages for fractal kinetics is faster than it was at the same window of time for second-order and first-order, a result that is a manifestation to some extent of the additive character of the two different orders.

As a practical application of the models listed in Table 1, we determine the type of adsorption of two experimental processes reported in the literature.^47,48^ The first study considers the removal of arsenic from groundwater using crystalline hydrous ferric oxide (AS/HFO).^47^ The second study considers the adsorption of phosphate on iron hydroxide–eggshell waste.^48^ An open-access plot digitizer, https://digitizer.starrydata.org/, was used to obtain the numerical data of the two studies.

Four models used in Figure 5, matched the data for *C*_0_ = 50 mg L^–1^, with correlation coefficients ranging from 0.996 to 0.998. Notice that for fitting the data with first-order fractional kinetics, we used the numerical evaluation of the Mittag-Leffler function,^49^ and accordingly we used the function provided in MATLAB Central.^50^ The fit parameters are presented in Table 2. The best fit will be indicated by the distribution of errors (right panel of Figure 5) for each of the accepted models. Compared to all other models, first-order fractional modeling performs better across all experiment time scales.

**Figure 5:**
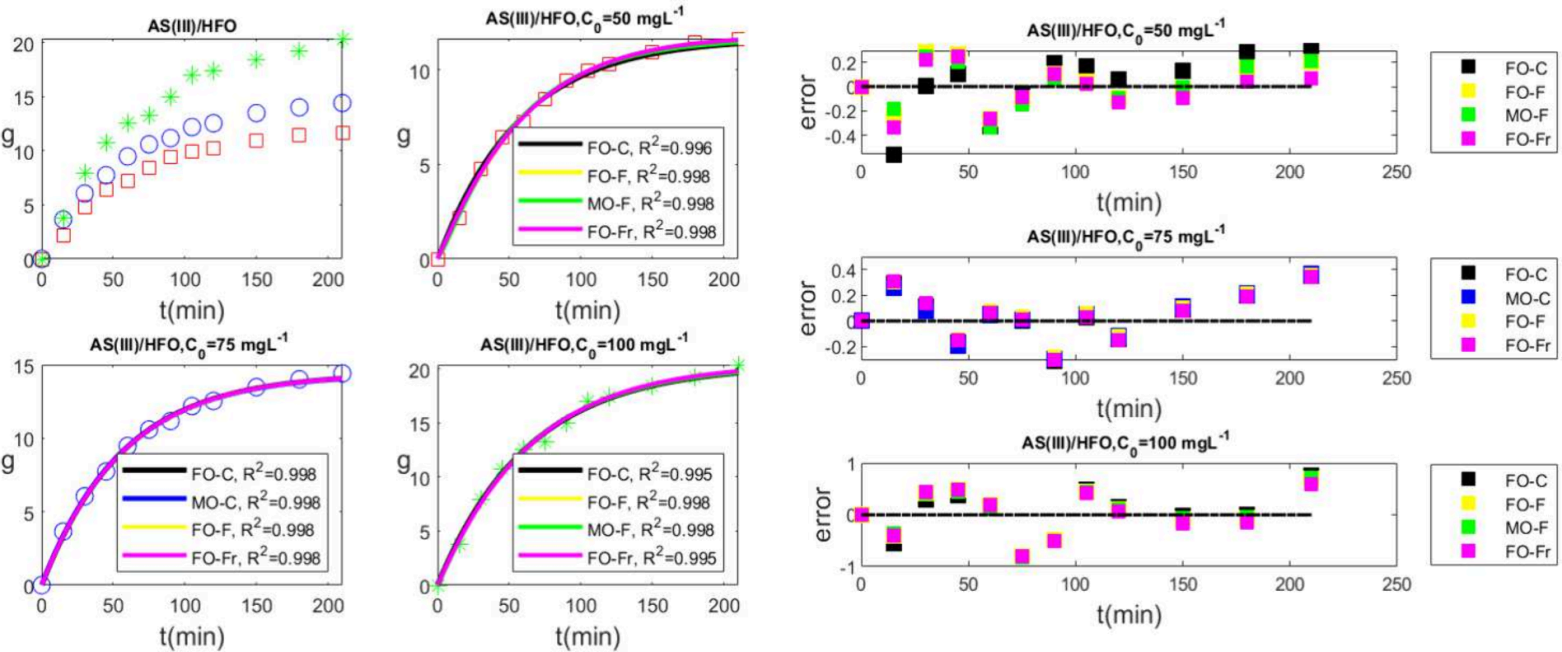
Removal of arsenic using crystalline hydrous ferric oxide for three different initial concentrations of As. Red for 50 mg L^–1^, blue for 75 mg L^–1^, and green for 100 mg L^–1^. Top left, experimental data, bottom left and top and bottom center, best fits with four different models, namely, FO-C, first-order classic; FO-F, first-order fractal; MO-F, mixed-order fractal; and FO-Fr, first-order fractional. Right, errors of the fits.

**Table 2:** Fit Parameters and Correlation Coefficients of Figure 5 ^a^

	AS(III)/HFO						
*C*_0_	FO-C	SO-C	MO-C	FO-F	SO-F	MO-F	FO-Fr
50 mL L^–1^	*k* = 0.017	*k* = 0.003	N.I.	*k* = 0.012	*k* = 0.0001	*k*_1_ = 0.006	*k* = 0.014
				α = 1.09	α = 1.74	*k*_2_ = 0.0002	α = 1.04
						α = 1.20	
*R* ^2^	0.996	0.919		0.998	0.991	0.998	0.998
75 mL L^–1^	*k* = 0.018	*k* = 0.002	*k*_1_ = 0.017	*k* = 0.018	*k* = 0.0002	N.I.	*k* = 0.015
			*k*_2_ = 0.00005	α = 0.998	α = 1.57		α = 1.00
*R* ^2^	0.998	0.938	0.998	0.998	0.987		0.998
100 mL L^–1^	*k* = 0.016	*k* = 0.001	N.I.	*k* = 0.014	*k* = 0.0001	*k*_1_ = 0.01	*k* = 0.014
				α = 1.02	α = 1.60	*k*_2_ = 0.015	α = 1.02
						α = 1.07	
*R* ^2^	0.995	0.931		0.995	0.987	0.995	0.995

a Notice that the proper units of the rates are 1/*T* for *k* of FO-C and *k*_1_ of MO-C, 1/*T* ^α^ for *k* of FO-F, FO-Fr, and *k*_1_ of MO-F, g/(mg *T*) for *k* of SO-C and *k*_2_ of MO-C, and g/(mg *T* ^α^) for *k* of SO-F and *k*_2_ of MO-F. The acronym N.I. means non-indicative fit.

As seen in Table 2, the calculated exponent for FO-Fr, α = 1.04, is close to the value of 1 at which the fractional kinetics returns FO-C. Fractal kinetics have an exponent of α = 1.09 and behaves similarly to fractional kinetics. Because the scaling exponent for both fractional and fractal kinetics is somewhat higher than the value of 1, and because there are not many observations, we can assign the adsorption kinetics to most straightforward classical mechanism. The assignment becomes clear for *C*_0_ = 75 mg L^–1^. Once more, four models (FO-C, MO-C, FO-F, and FO-Fr) fit the data with correlation coefficients ranging from 0.996 to 0.998. First-order fractal kinetics and first-order fractional kinetics return scaling exponents 0.998 and 1 (see Table 2), respectively, confirming thus first-order classical kinetics as the mechanism describing adsorption. For *C*_0_ = 100 mg L^–1^, the situation is unchanged; the best fitted models had a correlation coefficient of 0.995. We continue to use first-order classical kinetics as the adsorption mechanism because the scaling exponents of fractional and fractal kinetics have been calculated to be 1.02, which is extremely near to 1. Our results are consistent with the findings of the study that the adsorption process follows first-order classical kinetics, also known as the first-order Lagergren kinetic model.^47^ We should notice that a revised pseudo-second-order model has been applied to the same data sets.^51^

The second investigation considers the adsorption of phosphate on iron hydroxide–eggshell waste.^48^

Experimentally, see Figure 6, there is a quick initial phosphate absorption, followed by a progressive decrease of the rate. The time to reach the plateau depends on the initial concentration of the phosphate. These two features underline the existence of an adsorption mechanism that is more intricate than first-order classical kinetics.

**Figure 6:**
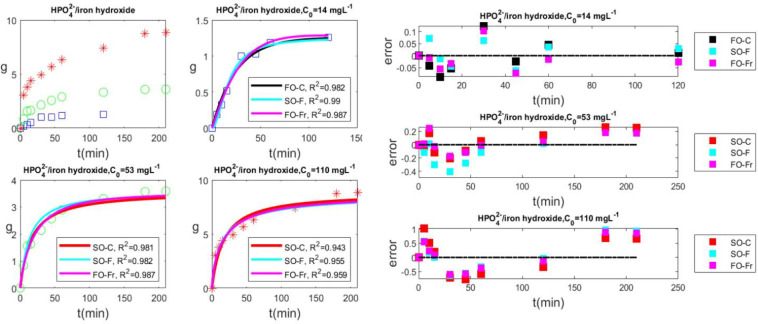
Adsorption of phosphate on iron hydroxide–eggshell waste for three different initial phosphate concentrations. Blue for 14 mg L^–1^, green for 53 mg L^–1^, red for 110 mg L^–1^. Top left, experimental data, bottom left and top and bottom center, best fits with three different models, namely, FO-C, first-order classic; SO-F, second-order fractal; and FO-Fr, first-order fractional. Right, errors of the fits.

Table 3 shows that FO-C kinetics has the poorest fit at initial concentrations of 53 and 110 mg L^–1^. The FO-C correlation coefficient for 14 mg L^–1^ is lower than that of first-order fractional kinetics and second-order fractal kinetics. For this system, FO-C kinetics is therefore disregarded as an adsorption mechanism. The model with the best correlation coefficient is associated with first-order fractional kinetics at all concentrations. Notice that similar or even better correlation coefficients appear for second-order fractal kinetics at the lowest concentrations. First-order fractional kinetics, second-order fractal kinetics, and second-order classical kinetics are the models that best describe the data. The errors illustrated in Figure 6 (right panel) indicate that the FO-Fr kinetics is the best model for describing adsorption of phosphate on iron hydroxide. Fractal kinetics assumes the presence of memory. In practice, the fits indicate that the phosphate molecules remain on the surface for a while before being adsorbed. The adsorption is not an on–off event. Surface heterogeneity or repulsion by other species present on the surface are factors that can chemically lead to this behavior. The present findings align with the conclusions of the experimental paper, which indicated that a pseudosecond-order kinetic model, followed by an intraparticle diffusion model^48^ or a revised pseudosecond-order model,^51^ were in operation.

**Table 3:** Fit Parameters and Correlation Coefficients of Figure 6 ^a^

	HPO_4_^2–^/iron hydroxide						
*C*_0_	FO-C	SO-C	MO-C	FO-F	SO-F	MO-F	FO-Fr
14 mL L^–1^	*k* = 0.040	*k* = 0.053	N.I.	N.I.	*k* = 0.004	N.I.	*k* = 0.03
					α = 1.89		α = 1.08
*R* ^2^	0.982	0.912			0.99		0.987
53 mL L^–1^	k = 0.034	k = 0.018	N.I.	N.I.	k = 0.018	N.I.	*k* = 0.068
					α = 1.07		α = 0.82
*R* ^2^	0.916	0.981			0.982		0.987
100 mL L^–1^	*k* = 0.03	*k* = 0.007	N.I.	N.I.	*k* = 0.012	N.I.	*k* = 0.01
					α = 0.82		α = 0.69
*R* ^2^	0.845	0.943			0.955		0.959

a Units, see Table 1.

## Conclusion

Classical kinetic modeling, which is the use of classical integer order derivatives, offers solutions that may reproduce experimental evidence. If the rate at which the material is adsorbed is proportional to some power of the material that has already been adsorbed, the adsorption-limited kinetics can be modeled using pseudo-order models of various orders. Fractal kinetics is a good approach for interpreting experimental data when there is a breakdown of the requirement of a homogeneously uniform distribution of the adsorbing material or when the geometry of the environment causes the free solute molecules to localize. Fractional kinetics is most likely the best method for comprehending adsorption kinetics when the conditions that lead to classical or fractal kinetics are not met. It also introduces memory that may affect a limited number of future events, as in the case of intraparticle diffusion.^16^ We have presented a few straightforward examples from which it is evident the diverse kinetics described by the different modeling (classical, fractal, or fractional) result in truly different trends.
